# Molecular Docking of Cissus quadrangularis and Allium sativum Against Pilus Islet-2 of Streptococcus oralis: An In Silico and In Vitro Analysis

**DOI:** 10.7759/cureus.98165

**Published:** 2025-11-30

**Authors:** Janani Priya K S, Renuka Devi R, Kokila G, Nitya Kala, Sonika S, Vignesh T

**Affiliations:** 1 Department of Periodontics, KSR Institute of Dental Science and Research, Tiruchengode, IND

**Keywords:** allium sativum, antibacterial, antioxidant, cissus quadrangularis, in silico, molecular docking, pilus islet-2

## Abstract

Introduction: Molecular docking is a key computational method to predict interactions between small molecules and proteins. This study evaluated the antibacterial potential of *Cissus quadrangularis* and *Allium sativum* against *Streptococcus oralis* using both in silico and in vitro approaches.

Methods: Plant extracts were prepared via Soxhlet extraction using petroleum ether and analysed through gas chromatography-mass spectrometry. Molecular docking studies revealed that 3-deoxy-D-mannoic lactone from *Allium sativum* (binding affinity: -4.9 kcal/mol) and 1,4,7,10,13,16-hexaoxacyclononadecane from *Cissus quadrangularis* (binding affinity: -5.0 kcal/mol) exhibited strong binding affinities with the Pilus islet-2 protein of *Streptococcus oralis*, indicating potential interference with bacterial adhesion and virulence. The active compounds were isolated using thin-layer chromatography and high-performance liquid chromatography.

Results: In vitro antibacterial assays demonstrated inhibition zones of 12.85 ± 0.21 mm for *Allium sativum* and 12.6 ± 0.14 mm for *Cissus quadrangularis*. Antioxidant activity, assessed by DPPH assay, showed lower IC₅₀ values for *Allium sativum* (77.21 µg/ml) compared to *Cissus quadrangularis* (84.41 µg/ml).

Conclusion: These findings underscore the potential of 3-deoxy-D-mannoic lactone as a potential antimicrobial candidate against *Streptococcus oralis,* contributing to novel therapeutic strategies for periodontitis.

## Introduction

Periodontitis is a chronic inflammatory disease caused by microbial-host interactions, leading to periodontal tissue destruction. Its initiation is linked to biofilm formation, with *Streptococcus oralis*, an early coloniser, adhering to the acquired pellicle and co-aggregating with late pathogens like Porphyromonas gingivalis and Fusobacterium nucleatum. Pilus islet-2 (PI-2) serves as a key adhesion factor facilitating biofilm maturation and interspecies aggregation [1]. Targeting specific adhesins like PI-2 offers a sustainable strategy over broad-spectrum antibiotics, reducing antibiotic resistance and side effects [2]. Phytoconstituents such as phenolics and flavonoids interfere with streptococcal adhesion and extracellular polymeric substance synthesis, disrupting the early stages of oral biofilm development [3]. Essential oil from *Thymus vulgaris* has demonstrated bacteriostatic and bactericidal effects on *S. oralis*, significantly reducing its biofilm formation in vitro [4]. Similarly, extracts of *Allium sativum* exhibit inhibitory effects against oral streptococci, including *S.* oralis, isolated from dental plaque samples [5]. This study explores phytoconstituents from biocompatible, multi-targeted plant sources such as *Allium sativum *and *Cissus quadrangularis *as potential alternatives for precision-based periodontal therapy [6]. *Allium sativum* (garlic) is valued in Ayurveda, Unani, and Traditional Chinese Medicine for its antimicrobial, anti-inflammatory, and immunomodulatory properties, mainly due to allicin [7]. *Cissus quadrangularis* (“Hadjod”) is traditionally used for bone healing, inflammation, and gastrointestinal disorders, containing bioactive compounds like ketosteroids and flavonoids with antibacterial, antioxidant, and tissue-regenerative properties [8].

Molecular docking has increasingly become a valuable tool for predicting interactions between phytocompounds and oral microbial targets. Recent computational studies have shown that essential-oil constituents, including eugenol derivatives, possess favourable binding affinities with key virulence-associated proteins such as Sortase A, Sortase B, and glucan sucrase, indicating their potential relevance in oral disease modulation [9]. Further in silico investigations have demonstrated that naturally occurring flavonoids also exhibit meaningful protein interactions, with luteolin displaying strong affinity for Cyclin D (−5.45 kcal/mol) and chrysoeriol for the PI3K-RAS binding domain (−4.60 kcal/mol) [10]. Building on this evidence, the present study explores the bioactive constituents of *Cissus quadrangularis* and *Allium sativum *against the PI-2 protein of *S. oralis* to identify potential inhibitory candidates.

While recent in silico studies in oral microbiology have effectively used molecular docking to prioritise phytocompounds for experimental testing, many investigations conclude with crude-extract antimicrobial assays or rely solely on docking predictions [11]. To bridge this gap, the present study targets the PI-2 adhesin of *S. oralis* and employs an integrated workflow encompassing docking, compound isolation, and in vitro validation. This structured approach enables clear identification of active phytochemicals and strengthens the precision and translational relevance of periodontal drug-discovery research.

The study pursues the following objectives: 1) To analyse the putative hits based on the highest binding affinity of *Cissus quadrangularis* and *Allium sativum* against PI-2 of *S. oralis *through molecular docking and 2) To evaluate the antibacterial and antioxidant efficacy of the active compounds of *Cissus quadrangularis* and *Allium sativum* against *S. oralis*.

The study promotes a plant-based approach with minimal adverse effects as a sustainable alternative to broad-spectrum antibiotics.

## Materials and methods

Study design

This in silico and in vitro study was presented to the Institutional Review Board-KSRIDSR for protocol approval, and ethical clearance was obtained from the Institutional Ethics Committee of KSR Institute of Dental Science and Research (IEC Ref No: 387/KSRIDSR/IEC/2023). *Cissus quadrangularis *stem and *Allium sativum* pod were collected from areas in and around Salem, Eastern Ghats of Tamil Nadu, shade-dried, and ground into fine powder. Streptococcus oralis-2696 was procured from the Microbial Type Culture Collection (MTCC), Chandigarh, India.

Soxhlet extraction of plant compounds

**Figure 1:**
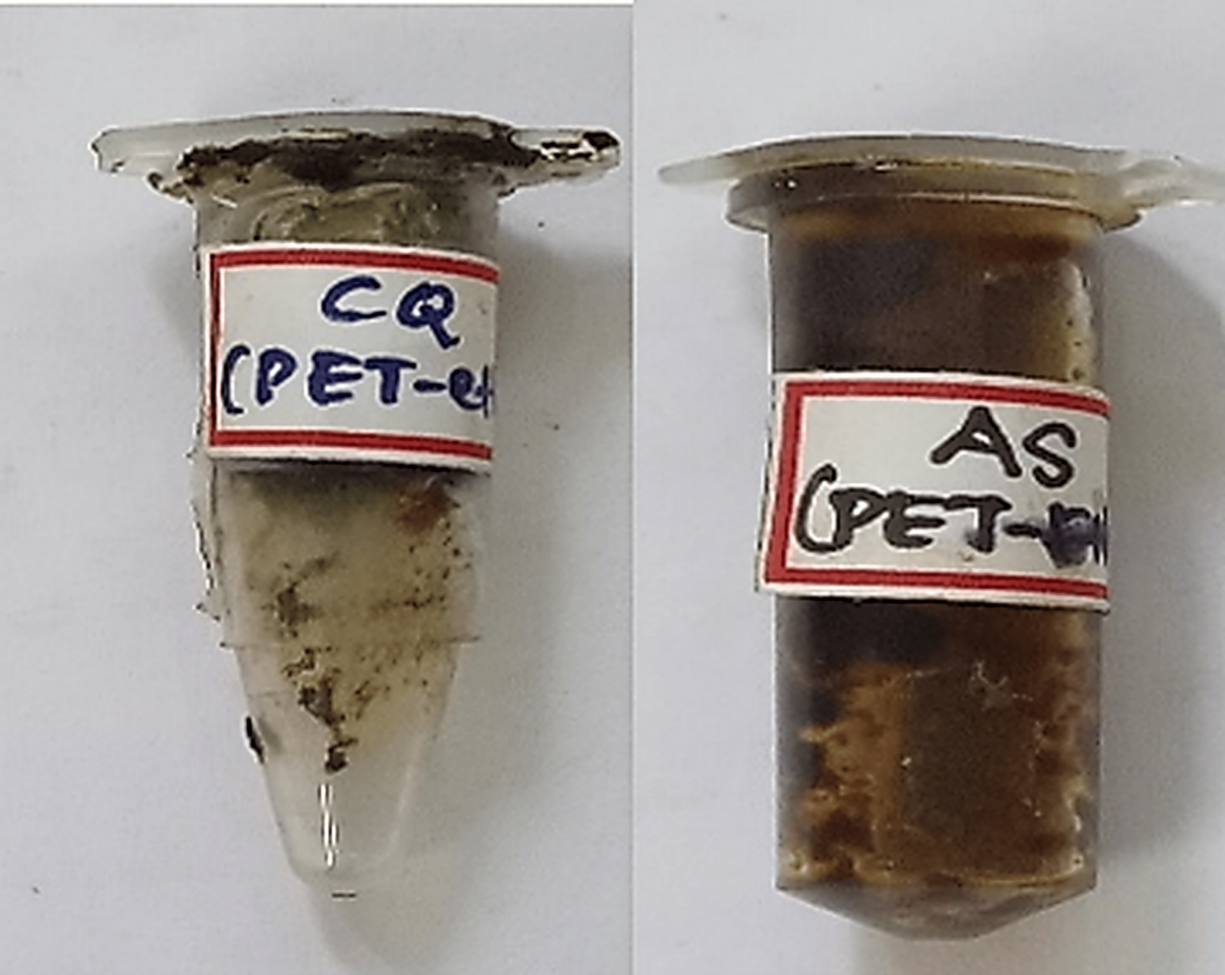
Extract of Cissus quadrangularis (Cq) and Allium sativum (As)

The powdered plant materials of *Cissus quadrangularis* (Cq) and *Allium sativum* (As) were subjected to Soxhlet extraction using petroleum ether (PET-ether) as the solvent. Fifty grams of each powdered sample were placed in a cellulose thimble within the Soxhlet apparatus, and 100 mL of PET-ether was used for continuous extraction over four hours [12]. The extracts were concentrated by solvent evaporation using a rotary evaporator under reduced pressure, then collected and stored in sterile containers at 4°C for further analysis (Figure 1).

Identification of phytochemical compounds using gas chromatography-mass spectrometry (GC-MS) analysis

A Shimadzu GC-QP2010SE system with an AOC-20i autosampler (Shimadzu Corporation, Kyoto, Japan) was used for GC-MS analysis under optimized conditions for phytochemical profiling. The prepared samples (Cq and As) were injected in 1.00 µL aliquots after derivatization to enhance thermal stability and volatility. Split mode injection (10:1) was performed at 250°C, with three solvent pre- and post-rinses, high plunger speed, five pumping cycles, and a syringe cleaning volume of 6 µL. The column oven temperature started at 50°C, increased to 280°C at 6°C/min, and was held for two minutes; helium was used as the carrier gas with flows optimized for capillary column separation [13]. Mass spectrometry was performed at interface and ion source temperatures of 200°C and 250°C, with a solvent cut-off of 3.50 minutes, a detection range of 50-500 m/z, and data captured at 1666 scans/sec. Phytochemical peaks were analyzed against the NIST20R library [14], and quantitative data from peak heights and areas enabled accurate, reproducible profiling.

Molecular docking using AutoDock 

The bioactive compounds identified from GC-MS were obtained from the PubChem library for Cq and As for docking studies. Ligand preparation was done using BIOVIA Discovery Studio and AutoDock Tools 4.2.6 [15,16]. The target protein, Pilus islet-2 adhesion protein (PitA) of Streptococcus oralis (PDB ID: 7W6B), was refined for docking. Docking simulations were performed using AutoDock Vina. The grid box was centred at X = −11.66, Y = 68.90, and Z = −1.71 with dimensions 24 × 24 × 24 Å, fully covering the binding pocket. An exhaustiveness of 8, 9 output modes, and 10 independent docking runs were used for each ligand. For validation, the native ligand was redocked into the binding site and reproduced the experimental pose with an RMSD of ~1.7 Å, confirming the reliability of the docking protocol. The structural preparation of ligands and the target protein was carried out using AutoDock 4.2.6, whereas the molecular docking simulations were performed using AutoDock Vina.

Separation of active compounds by column chromatography

Crude extracts of Cq and As were subjected to column chromatography using silica gel (60-120 mesh) packed by the wet method with methanol, with extracts premixed with silica and layered onto the column [17]. Elution was performed using a stepwise gradient: 100% ethanol, 1:1 ethanol: n-hexane, and 100% n-hexane. Fractions were collected in 10 mL volumes and monitored for compounds via TLC analysis.

Profiling of plant extracts using thin layer chromatography (TLC)

TLC was used to examine phytochemical components of petroleum ether extracts from *Cissus quadrangularis* and *Allium sativum *on pre-coated silica gel plates (stationary phase) with samples spotted 0.5 cm above the baseline and mobile phase toluene: ethyl acetate: formic acid (5:4:0.2) [18]. Plates were developed in a pre-saturated chamber, dried, and visualised under UV light at 254 nm and 366 nm.

Characterisation of extracts using high-performance liquid chromatography (HPLC)

HPLC was performed using a THAVA HPLC system with a UV detector. Reverse-phase chromatography was employed with an appropriate mobile phase and detection wavelengths of 210-370 nm [19]. Flow rates and run times were optimized for efficient separation and identification of active compounds.

Antibacterial activity of the putative hits of Cq and As

**Figure 2:**
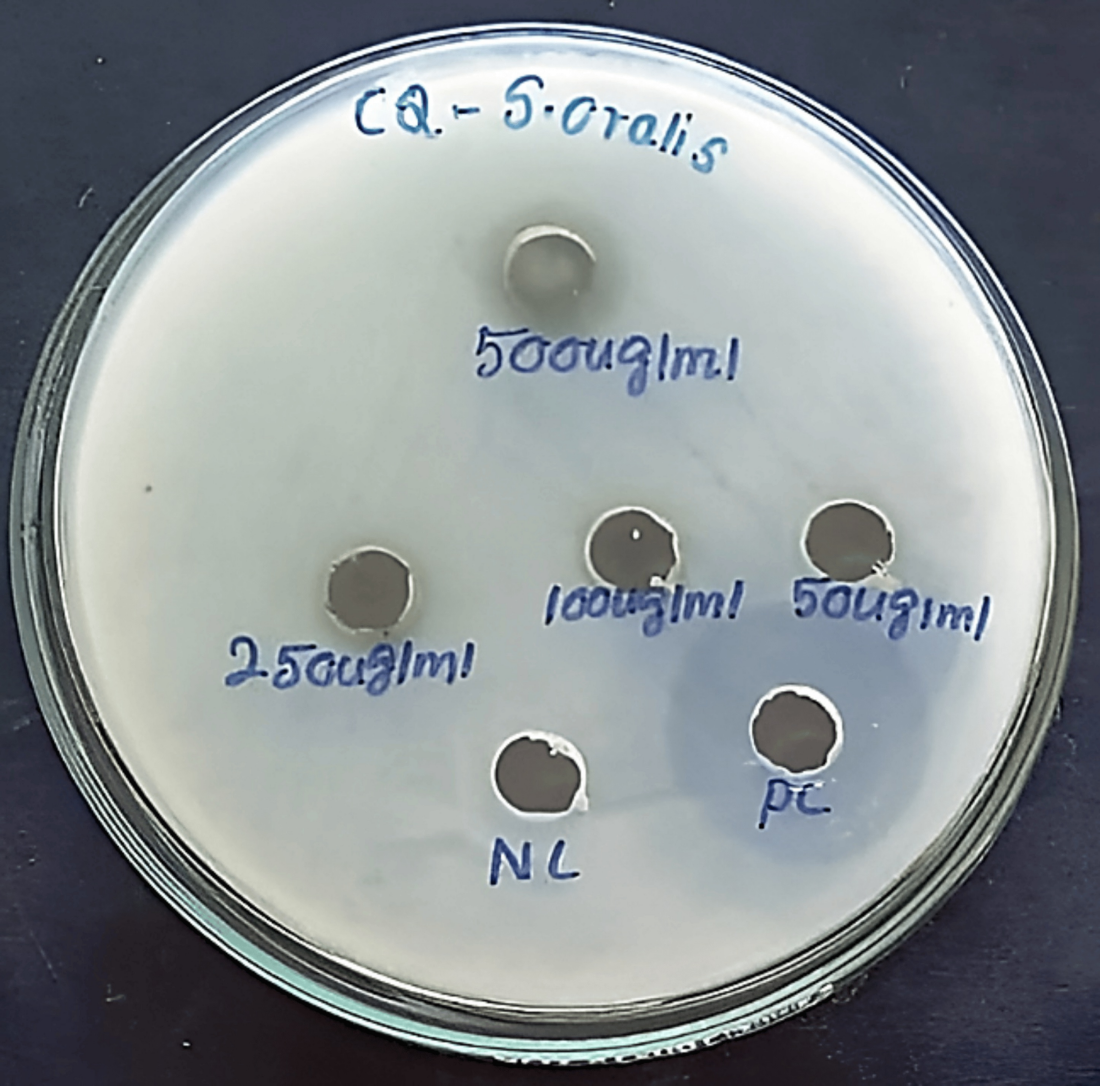
Zone of inhibition of 1,4,7,10,13,16-hexaoxacyclononadecane of Cissus quadrangularis (CQ) against S. oralis. 50-500 micrograms per millilitre indicates the concentration of the extract. Positive control (PC): Gentamicin, Negative control (NC): Dimethyl sulfoxide

**Figure 3:**
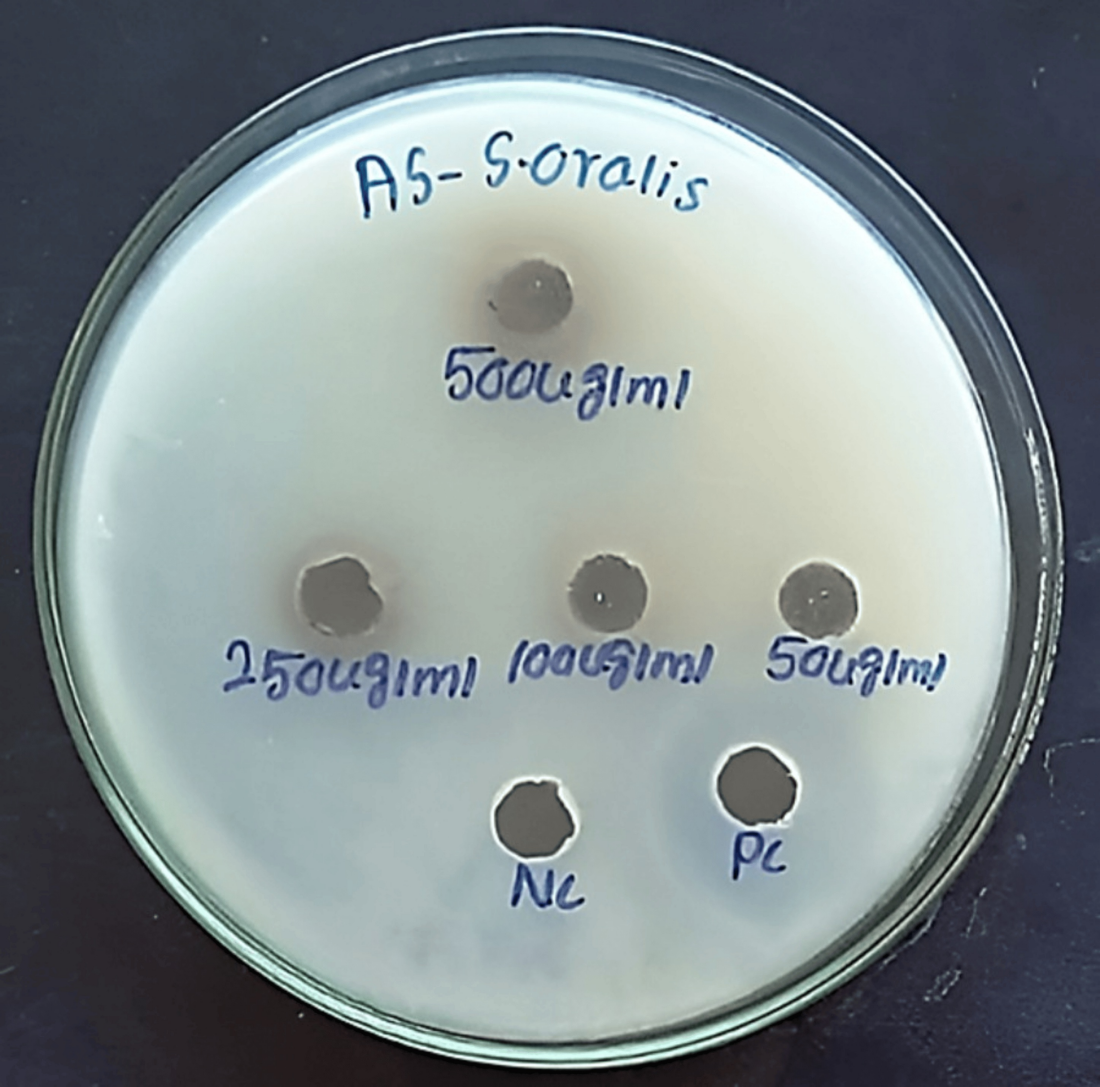
Zone of inhibition of 3-deoxy-d-mannoic lactone of Allium sativum (AS) against S. oralis. 50-500 micrograms per millilitre indicates the concentration of the extract. Positive control (PC): Gentamicin, Negative control (NC): Dimethyl sulfoxide

Purified active compounds of Cq and As were evaluated against Streptococcus oralis (MTCC-2696) using the agar well diffusion method [20]. Test concentrations of active compounds of Cq and As at 500, 250, 100, and 50 µg/mL were used, with Gentamicin (20 µg/mL) as a positive control. Plates were incubated at 37°C for 24 hours, and clear zones around each well indicate inhibition of bacterial growth. The diameter of the zone of inhibition (in mm) was measured in millimetres. Experiments were performed in triplicate for reproducibility (Figures 2, 3).

Antioxidant activity of the putative hits of Cq and As

Antioxidant activity was assessed using the DPPH (2,2-diphenyl-1-picrylhydrazyl) radical scavenging assay. Test samples (500-10 µg/mL) were mixed with DPPH and incubated in the dark for 30 minutes, and absorbance was measured at 517 nm [21,22], with ascorbic acid as the standard. % Radical scavenging was calculated as % Inhibition = [(Absorbance of control − Absorbance of sample) / Absorbance of control] × 100.

Statistical analysis

Data were analyzed using IBM SPSS Statistics for Windows, Version 22 (Released 2022; IBM Corp., Armonk, New York, United States) and GraphPad Prism 6.0 (GraphPad Software, San Diego, CA). Mean and SD were calculated for antibacterial zones and antioxidant inhibition. The Kruskal-Wallis test tested antibacterial differences; one-way ANOVA with Tukey’s HSD tested antioxidant activity. IC₅₀ was estimated by regression, with significance at p < 0.05.

## Results

Phytochemical screening using GC-MS analysis

All chromatogram peaks were examined and authenticated against the NIST20R library for accurate chemical identification. In *Cissus quadrangularis* (Cq), the most prominent compound was decaethylene glycol (TMS derivative), with a retention time of 34.766 min, a peak area of 2,281,608, and a height of 261,502, indicating high abundance and enhanced volatility for GC-MS analysis. In *Allium sativum* (As), glycerin (1,2,3-propanetriol) was the major component, with a retention time of 6.565 min, peak area of 20,135,878, and height of 525,838, showing a higher quantity than other compounds. Decaethylene glycol and other TMS derivatives highlight Cq’s potential as a source of bioactive substances. Glycerin’s hydrophilic nature and metabolic role are important in garlic’s biochemical and therapeutic effects (Tables 8, 9 in the Appendices).

In silico docking analysis

**Figure 4:**
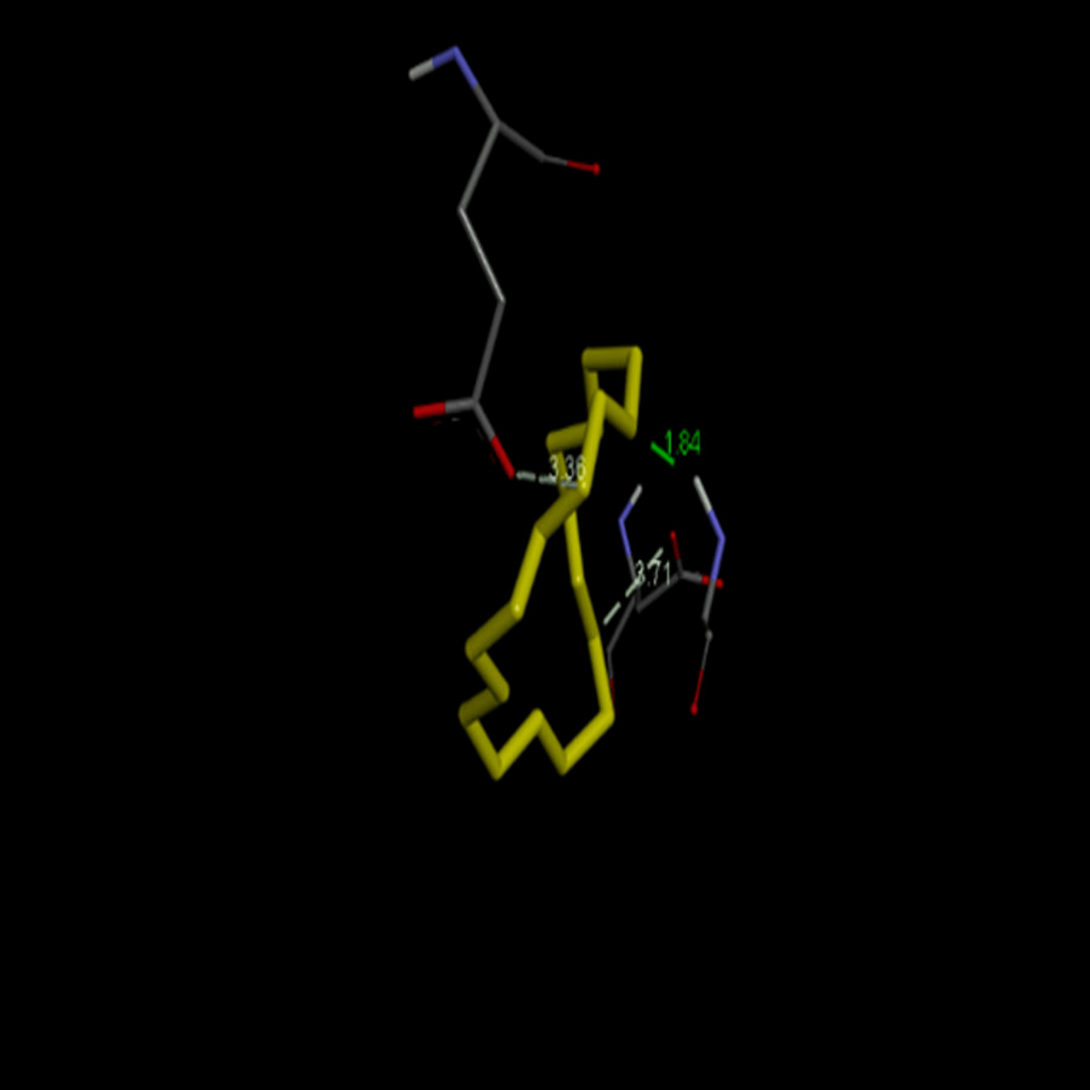
Interaction of PitA - Streptococcus oralis (yellow) with 1,4,7,10,13,16-hexaoxacyclononadecane (grey)

**Figure 5:**
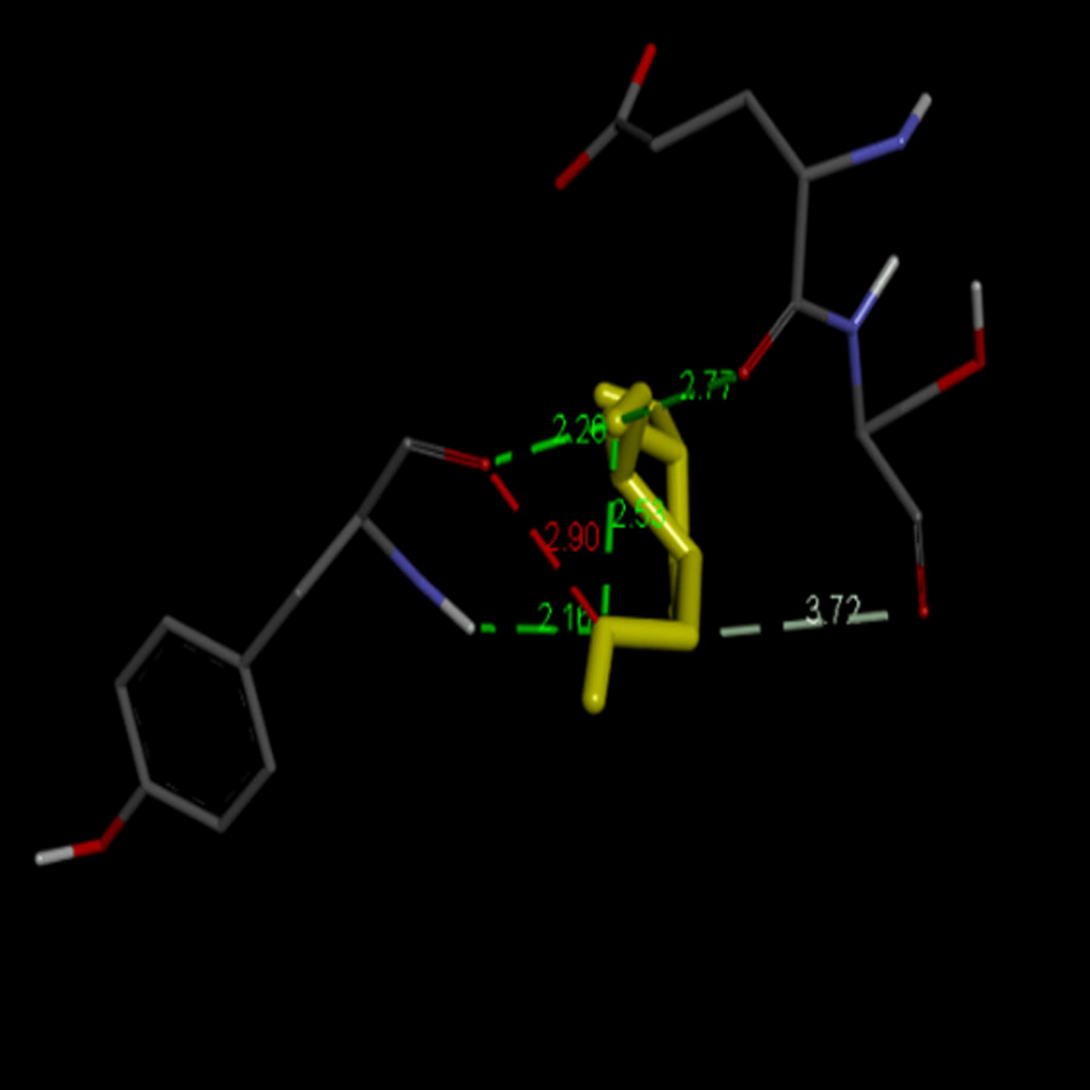
Interaction of PitA - Streptococcus oralis (yellow) with 3-deoxy-d-mannoic lactone (grey)

The chemical structure of PitA - Streptococcus oralis was analysed with 1,4,7,10,13,16-hexaoxacyclononadecane and 3-deoxy-D-mannoic lactone (Figures 4, 5). Docking of PitA (7W6B) with 1,4,7,10,13,16-hexaoxacyclononadecane from Cq showed the highest binding affinity of -5.0 kcal/mol, while 3-deoxy-D-mannoic lactone from As exhibited -4.9 kcal/mol (Table 1). This study molecularly docked the top-scoring compounds from Cq and As against the PI-2 of *S. oralis*.

**Table 1:** Binding affinities of the putative hits of Cq and As

S.no	Bonds	Binding Affinity	Amino Acid Residues
7W6B with 1,4,7,10,13,16-Hexaoxacyclononadecane from Cissus quadrangularis	5 Hydrogen	-5.0 Kcal/mole	A:GLY569:HN - :UNK0:O6 - 1.83 Å
	Conventional Hydrogen Bond		:UNK0:C9 - A:ASP57:OD2 – 3.07 Å
	Carbon-Hydrogen Bond		:UNK0:C18 - A:GLU592:OE2– 3.36 Å
				7W6B with 3-Deoxy-d-mannoic lactone from Allium sativum	5 Hydrogen bonds	-4.9 Kcal/mole	A:TYR570:HN - :UNK0:O4 - 2.14 Å
Conventional Hydrogen Bond	:UNK0:H19 - A:TYR570:O – 2.25 Å					
Carbon-Hydrogen Bond	:UNK0:H19 - A:GLU592:O – 2.76 Å					
	:UNK0:H19 - :UNK0:O4 – 2.53Å					
	:UNK0:C10 - A:SER593:O – 3.72 Å					

TLC analysis

**Figure 6:**
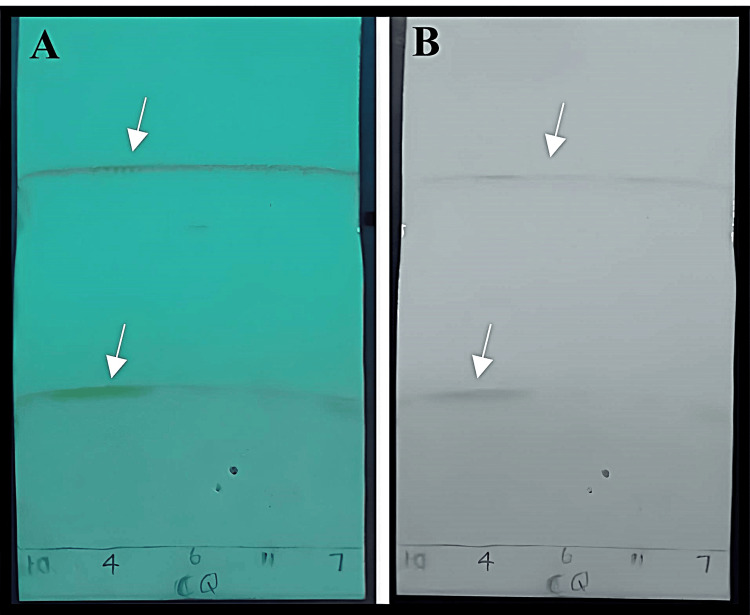
Band pattern of Cissus quadrangularis Panel A: UV light, Panel B: Visible light. White arrows indicate separated phytochemical compounds; the numbered spots 4, 6, 7, 10, and 11 indicate different solvent ratios tested of Cq

**Figure 7:**
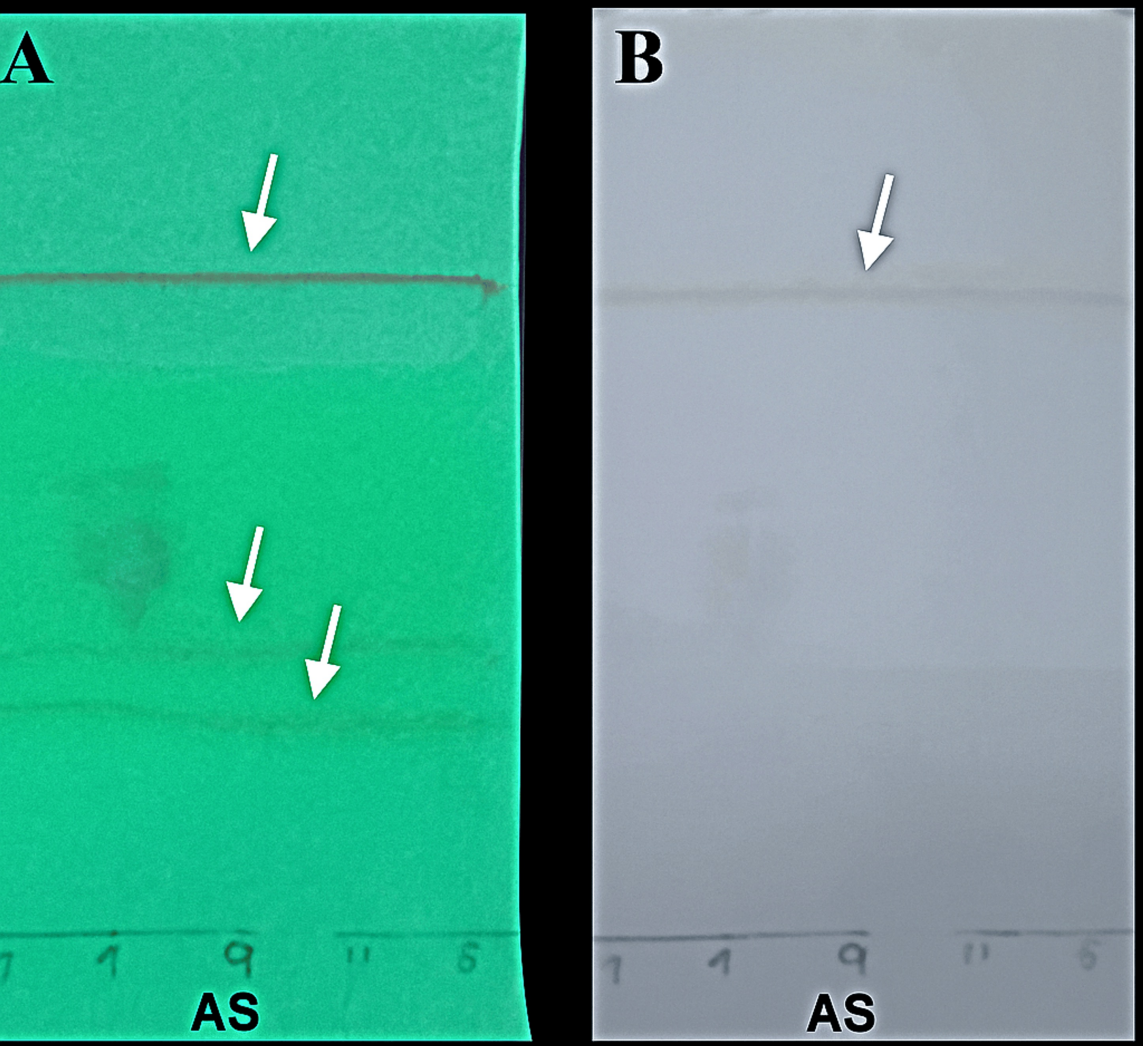
Band pattern of Allium sativum Panel A: UV light, Panel B: Visible light. White arrows indicate separated phytochemical compounds; the numbered spots 7, 4, 9, 11, and 6 indicate different solvent ratios tested of As extracts

TLC analysis (Table 2) showed that *Cissus quadrangularis* contained moderately polar phytoconstituents with retention factor (Rf) values of 0.51-0.56, while *Allium sativum* had more non-polar molecules with Rf values of 0.67-0.76. Rf is calculated as compound travel distance ÷ solvent front travel distance, ranging from 0 to 1, with higher values indicating greater solubility in the mobile phase. Lower Rf values indicate a stronger attraction to the stationary phase. A TLC plate visualised under UV light at 254 nm showing separated phytochemical bands (Panel A). The same plate was visualised under visible light after spraying with iodine vapour for compound visualisation (Panel B). The numbered spots (4, 6, 7, 10, 11) and (7, 4, 9, 11, 6) indicate different solvent ratios tested of Cq and As extracts, respectively. Distinct band patterns under UV light and visible light confirmed multiple bioactive compounds in both extracts (Figures 6, 7). 

**Table 2:** Rf values of Cq and As

Name of Test Samples	Rf Values
CQ-10	0.51
CQ-4	0.51
CQ-6	0.56
CQ-11	0.55
CQ-7	0.53
AS-7	0.74
AS-4	0.67
AS-9	0.68
AS-11	0.72
AS-6	0.76

HPLC analysis

**Figure 8:**
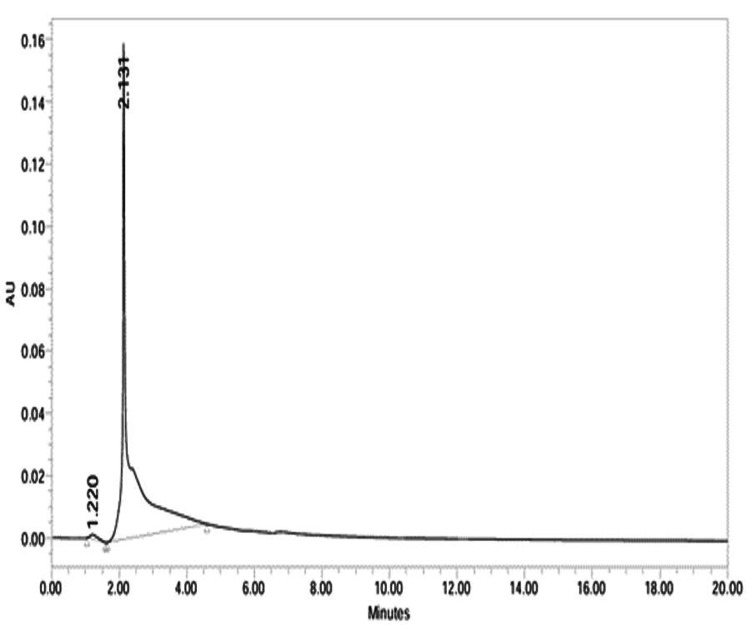
Chromatogram of 1,4,7,10,13,16-hexaoxacyclononadecane of Cq

**Figure 9:**
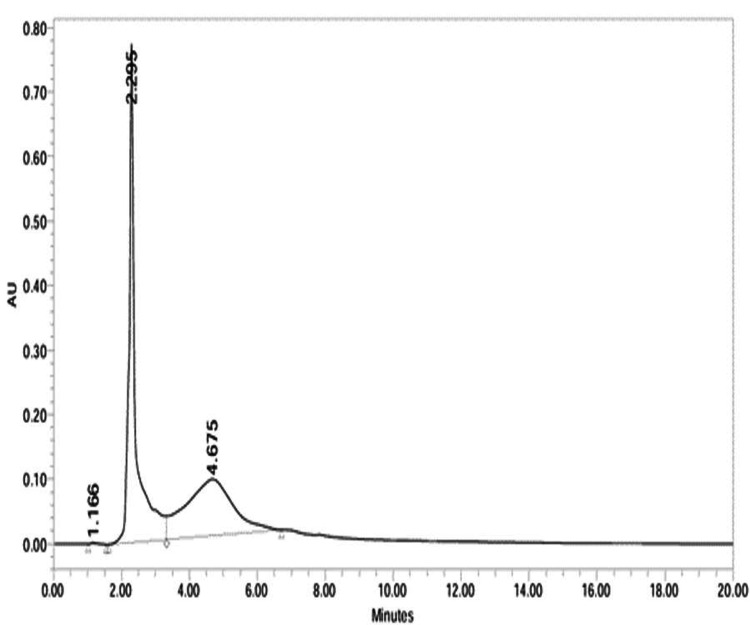
Chromatogram of 3-deoxy-d-mannoic lactone of As

Figure 8 showed two peaks for *Cissus quadrangularis*, with a major peak at 2.131 min (98.79%) identified as 1,4,7,10,13,16-hexaoxacyclononadecane. Figure 9 for *Allium sativum* showed three peaks, with 2.295 min (54.58%) and 4.675 min (45.26%) representing 3-deoxy-D-mannoic lactone. Identification was based on retention time, UV absorbance, and NIST20R library comparison, highlighting dominant phytochemical constituents.

In vitro antibacterial activity

At 500 µg/mL, the active compound of As showed the greatest inhibition (12.85 ± 0.21 mm) compared to Cq (12.6 ± 0.14 mm), and at 250 µg/mL, As (12.25 ± 0.35 mm) exceeded Cq (11.35 ± 0.21 mm). At 100 µg/mL, both showed moderate activity (8.35 mm), while at 50 µg/mL, no inhibition was observed, indicating a threshold of activity (Table 3). Compared to gentamicin (19.25 ± 0.35 mm), both extracts were weaker. P-values >0.05 suggest differences across concentrations were not statistically significant. 

**Table 3:** Mean ± SD of inhibition zones for the Active compounds of Cq and As SD: Standard Deviation; PC: Positive Control, *Significance: p< 0.05

Name of the Test Organism	Name of the Test Sample	Zone of Inhibition (mm)						
		Mean ± SD						
		500 µg/ml	250 µg/ml	100 µg/ml	50 µg/ml	PC	p-value	
S. oralis	1,4,7,10,13,16-Hexaoxacyclononadecane	12.6±0.14	11.35±0.21	8.35±0.21	0	19.25±0.35	0.405	
									3-Deoxy-d-mannoic lactone	12.85±0.21	12.25±0.35	8.35±0.49	0	17.1±0.14	0.405	

DPPH radical scavenging assay

The active compound of Cq showed 61.15% inhibition at 500 µg/mL, while ascorbic acid exhibited 74.17% (Appendix Tables 10, 11). ANOVA (F(5,12)=132.61, p<0.0001) and Tukey’s HSD confirmed significant differences between groups, with variations within Cq concentrations not significant (p>0.05) (Tables 6, 7 in the Appendices) and IC₅₀ of 84.41 µg/mL (Table 4).

**Table 4:** One-way ANOVA for antioxidant activity of 1,4,7,10,13,16-hexaoxacyclononadecane of Cq *P <0.0001  statistically significant

Source of Variation	SS (Sum of Squares)	df (Degrees of Freedom)	MS (Mean Square)	F	p-value
Between Groups	663.11	5	132.62	136.21	< 0.0001*
Within Groups	11.68	12	0.973		
Total	674.79	17			

For As, antioxidant activity ranged from 56.80% at 10 µg/mL to 65.20 % at 500 µg/mL, with ANOVA (F(5,12)=168.58, p<0.0001) showing significant differences among groups (Table 5). Ascorbic acid was significantly higher than all As concentrations (p<0.001), with an IC₅₀ of As at 77.21 µg/mL. These results indicate concentration-dependent antioxidant effects of Cq and As compounds.

**Table 5:** One-way ANOVA for antioxidant activity of 3-deoxy-d-mannoic lactone of As *p <0.0001  statistically significant

Source of Variation	SS	df	MS	F	p-value
Between Groups	464.54	5	92.91	168.58	< 0.0001*
Within Groups	6.61	12	0.5508		
Total	471.15	17			

## Discussion

Periodontal disease is a multifactorial condition involving complex interactions among microbial pathogens, host immune responses, genetic predispositions, and environmental factors [23]. While dental plaque has long been recognised as the primary etiological factor, its presence alone does not fully explain the disease's severity; instead, the host's immune reaction to microbial insult plays a critical role in tissue destruction. Consequently, targeting plaque formation serves a dual purpose: reducing microbial burden and mitigating the exaggerated host response [24].* S. oralis,* an early coloniser in plaque biofilms, adheres to tooth surfaces via pili structures, initiating plaque formation. Our study aimed to disrupt this initial adhesion by molecular docking of individual plant-derived phytocompounds from *Cissus quadrangularis* and *Allium sativum *against the PI-2 proteins of S. oralis, thereby interfering with its virulence mechanism.

Recent studies strongly support the therapeutic potential of plant-based medications in the prevention and treatment of periodontitis. A meta-analysis concluded that herbal mouth rinses often matched the clinical efficacy of chlorhexidine in reducing plaque and gingival inflammation, with markedly fewer side effects [25]. Specifically, *Allium sativum* (garlic) extract has been shown to upregulate anti-inflammatory cytokines, such as IL-10 and IL-13, which significantly reduce alveolar bone resorption in experimental periodontitis models [26]. Meanwhile,* Cissus quadrangularis* has garnered attention for its antibacterial activity against keystone periodontal pathogens. In vitro studies from 2024 demonstrated that ethanolic extracts of Cissus quadrangularis inhibited Porphyromonas gingivalis at MICs as low as 500 µg/mL, with an IC₅₀ of 194 µg/mL, highlighting its potential as a natural antimicrobial agent [27]. Furthermore, biomaterial research has incorporated *Cissus quadrangularis *into scaffolds that enhance periodontal tissue regeneration, showing excellent biocompatibility and osteogenic potential in bone defect models [28]. These findings provide a robust scientific foundation for exploring phytoconstituents from *Allium sativum* and *Cissus quadrangularis *as precision-targeted therapeutic agents against early colonisers like S. oralis. Such plant-derived interventions offer a sustainable alternative to conventional antibiotics, aiming to preserve the oral microbiome while mitigating resistance and side effects.

Focusing on isolated active compounds rather than crude extracts enhances pharmacological efficiency and reduces biological load. Using AutoDock software, this in silico approach offers a cost-effective strategy to identify potent anti-adhesion agents, guiding the development of novel chemotherapeutic agents for managing periodontal disease before progressing to labour-intensive in vitro or clinical trials [29].

Fan et al. (2023) demonstrated that a solid dispersion of Polygonum cuspidatum extract, rich in resveratrol, exhibited enhanced dissolution and oral bioavailability in rats, suggesting potential for improved in vivo antioxidant and anti-inflammatory activity [30]. Correspondingly, Dar et al. (2023) identified glycerol as a major component with antimicrobial synergy with sulfur-containing compounds of aqueous garlic extract [31].

In the present study, binding affinities for *Cissus quadrangularis *(Cq) and *Allium sativum* (As) were similar to those reported by Saini et al. (2024), who evaluated compounds like myricetin, rotenone, and chelidonine against heme-binding proteins of Porphyromonas gingivalis using AutoDock [32]. Targeting Streptococcus oralis, an early coloniser, offers a strategic advantage by disrupting initial biofilm formation, underscoring the preventive potential of Cq and As in halting periodontitis progression at its onset.

TLC of Cq revealed five components with Rf values of 0.51, 0.51, 0.56, 0.55, and 0.53, which were further standardised via HPLC to identify 4,7,10,13,16-hexaoxacyclonononadecane. Similarly, Garg et al. (2024) isolated 12 constituents from ethanolic Cq extract with Rf values ranging from 0.06 to 0.95 using TLC [33]. These results highlight dominant phytochemical constituents and align with Bhusari et al. (2020), demonstrating that dual-retention methods like HPLC effectively separate low- and high-polarity compounds in complex plant matrices [34].

The study by Jain et al. (2021), where whole plant extracts of *Allium sativum* demonstrated significant antibacterial effects against the oral pathogen *S. oralis*. This comparison suggests that the current study evaluated the efficacy of the isolated compound 3-deoxy-D-mannoic lactone from As as having equivalent antimicrobial potential to the whole plant extract of Allium sativum in targeting oral pathogens [35].

The antioxidant activity of the isolated phytocompounds from *Allium sativum* (As) and *Cissus quadrangularis* (Cq) was assessed using the DPPH free radical scavenging assay. The compound from *Allium sativum *showed comparatively higher antioxidant potential; however, the isolated compound from *Cissus quadrangularis* also demonstrated substantial free radical scavenging activity. Interestingly, this is in agreement with the findings of Murthy et al. (2003), where the ethyl acetate fraction of the whole Cissus quadrangularis extract exhibited similar antioxidant activity [36].

Limitations and future directions

The present study has several limitations. Firstly, it focused on a single bacterial protein target, which restricts the scope of microbial evaluation and does not encompass the diversity of periodontal pathogens. Secondly, the in vitro antibacterial and antioxidant assays were performed at limited concentration ranges, and more comprehensive concentration-response analyses are required to establish the dose-dependent efficacy of the tested compounds. Additionally, critical parameters such as cytotoxicity and biocompatibility were not assessed, both of which are essential for determining the safety and potential applicability of these phytochemicals in therapeutic contexts.

Future investigations should therefore focus on exploring the synergistic effects of these phytoconstituents when combined with conventional periodontal therapies. Further studies should also examine their potential influence on quorum-sensing mechanisms and quorum threshold behaviour within multispecies oral biofilm models. Moreover, extending the evaluation of these bioactive compounds through in vivo experiments, including animal studies and human clinical trials, would provide valuable insights into their real-world efficacy and safety as promising adjuvants in periodontal therapy.

## Conclusions

This study presents a novel strategy to prevent periodontal disease by targeting pili-mediated adhesion of *S. oralis*, inhibiting initial plaque biofilm formation. Phytochemical constituents from Cq and As offer targeted efficacy at reduced doses with lower risk of antimicrobial resistance compared to broad-spectrum antibiotics. The findings support the potential of these compounds as anti-adhesion agents. Thus, they may serve as adjuncts in periodontal therapy.

## Appendices

**Table 6:** Post hoc test: Tukey’s HSD of the active compound of As *p <0.001 statistically significant

Group Comparison	Mean Difference	p-value
Ascorbic vs Active compound of AS 10 µg/ml	17.45	<0.001*
Ascorbic vs Active compound of AS 50 µg/ml	16.07	<0.001*
Ascorbic vs Active compound of AS 100 µg/ml	11.31	<0.001*
Ascorbic vs Active compound of AS 250 µg/ml	10.21	<0.001*
Ascorbic vs Active compound of AS 500 µg/ml	9.06	<0.001*
Active compound of AS 500 µg/ml vs Active compound of AS 10 µg/ml	8.39	<0.001*
Active compound of AS 500 µg/ml vs Active compound of AS 250 µg/ml	1.15	>0.05
Active compound of AS 250 µg/ml vs Active compound of AS 100 µg/ml	1.1	>0.05

**Table 7:** Post hoc test: Tukey’s HSD of the active compound of Cq *p <0.001 statistically significant

Group Comparison	Mean Difference	p-value
Ascorbic vs Active compound of CQ 10 µg/ml	19.71	< 0.001*
Ascorbic vs Active compound of CQ 50 µg/ml	18.03	< 0.001*
Ascorbic vs Active compound of CQ 100 µg/ml	15.76	< 0.001*
Ascorbic vs Active compound of CQ 250 µg/ml	13.77	< 0.001*
Ascorbic vs Active compound of CQ 500 µg/ml	13.03	< 0.001*
Active compound of CQ 10 µg/ml vs Active compound of CQ 50 µg/ml	-1.68	> 0.05
Active compound of CQ 100 µg/ml vs Active compound of CQ 250 µg/ml	-1.99	> 0.05
Active compound of CQ 250 µg/ml vs Active compound of CQ 500 µg/ml	-0.74	> 0.05

**Table 8:** Bioactive compounds present in Cissus quadrangularis identified using GC-MS analysis GC-MS: Gas chromatography-mass spectrometry

Peak#	Area	R.Time	Height	Name
1	48579	6.019	16285	Cyclohexene, 1-methyl-4-(1-methylethenyl)-, (
2	42391	15.503	12627	Diethyl Phthalate
3	89058	15.644	21419	Phenol, 4-(1,1,3,3-tetramethylbutyl)-
4	205974	18.742	60269	Neophytadiene
5	36271	18.833	11804	1-Undecene, 9-methyl-
6	54927	19.061	13719	3,7,11,15-Tetramethyl-2-hexadecen-1-ol
7	67065	19.286	18991	3,7,11,15-Tetramethyl-2-hexadecen-1-ol
8	73251	20.265	22172	n-Decanoic acid
9	42267	21.872	12724	Decane, 2,3,7-trimethyl-
10	379727	22.058	108933	3,7,11,15-Tetramethyl-2-hexadecen-1-ol
11	100739	22.285	13356	1-Hexadecyne
12	119987	22.981	35874	Nonadecane
13	186169	23.858	23475	Diglycolic acid, 2-chloro-6-fluorophenyl unde
14	205321	23.947	47990	n-Nonadecanol-1
15	514517	24.051	119185	Heneicosane
16	881412	24.175	107677	18,18'-Bi-1,4,7,10,13,16-hexaoxacyclononadecane
17	762548	24.3	100895	18,18'-Bi-1,4,7,10,13,16-hexaoxacyclononade
18	253629	24.417	65609	Dodecane, 2,6,11-trimethyl-
19	423394	24.525	57389	Allyl n-octyl ether
20	962893	24.783	74527	1-Iodo-2-methylundecane
21	532803	24.869	78614	Tetratetracontane
22	1189744	25.072	185626	Heneicosane
23	617167	25.154	99703	Carbonic acid, eicosyl vinyl ester
24	1264050	25.421	97027	Tetrapentacontane, 1,54-dibromo-
25	396082	25.533	89178	3-Heptene, 2,2,3,5,5,6,6-heptamethyl-
26	916493	25.701	93490	Tetracosyl trifluoroacetate
27	1070444	25.783	94059	Sulfurous acid, octadecyl 2-propyl ester
28	2085142	26.057	186655	Octacosane, 2-methyl-
29	711944	26.392	66417	1-Hentetracontanol
30	836850	26.612	72298	Bis(2-ethylhexyl) phthalate
31	461961	27.008	87023	Heneicosane
32	160702	27.971	40709	Sulfurous acid, 2-propyl tridecyl ester
33	48316	29.105	12152	Nonane, 1-iodo-
34	2281608	34.766	261502	Decaethylene glycol, TMS derivative

**Table 9:** Bioactive compounds present in Allium sativum identified using GC-MS analysis GC-MS: Gas chromatography-mass spectrometry

Peak#	Area	R.Time	Height	Name
1	874260	3.765	129224	(S)-(+)-2-Amino-3-methyl-1-butanol
2	312136	4.132	93089	Disulfide, methyl 2-propenyl
3	621754	4.205	116567	Diisoamyl ether
4	118014	4.8	25519	3H-1,2-Dithiole
5	306499	5.042	37459	L-Proline, TMS derivative
6	244416	5.15	33170	2,4-Dihydroxy-2,5-dimethyl-3(2H)-furan-3-on
7	702150	5.356	112958	2-Hydroxy-gamma-butyrolactone
8	471639	5.423	109595	2H-Pyran-2,6(3H)-dione
9	1253436	5.543	163316	But-3-enyl (E)-2-methylbut-2-enoate
10	2E+07	6.565	525838	Glycerin
11	1114572	6.939	285542	Diallyl disulphide
12	315582	7.058	55519	Propargyl alcohol
13	882176	7.177	128817	3-(4-Bromophenyl)cyclobutan-1-amine, TMS
14	285334	7.32	74904	(E)-1-Allyl-2-(prop-1-en-1-yl)disulfane
15	534556	8.018	153993	Hydroperoxide, 1,4-dioxan-2-yl
16	203044	8.101	50185	4H-Pyran-4-one, 2,3-dihydro-3,5-dihydroxy-6
17	220640	8.575	27308	1-(Butylsulfanyl)-2-ethoxyethane
18	206371	8.712	36099	(S)-5-Hydroxymethyl-2[5H]-furanone
19	269083	8.915	52788	3-Vinyl-1,2-dithiacyclohex-4-ene
20	362103	9.095	84501	2-Amino-N,4-dimethylpentanamide
21	678937	9.354	78355	2-Vinyl-4H-1,3-dithiine
22	139502	9.475	29354	Benzene, 1-ethynyl-4-fluoro-
23	918059	9.635	139292	5-Hydroxymethylfurfural
24	162299	9.903	30451	Butanoic acid, 3-oxo-, hexyl ester
25	1030570	10.875	319155	Trisulfide, di-2-propenyl
26	193416	11.104	41106	2-Methoxy-4-vinylphenol
27	80589	11.342	20885	2-(Hydroxymethyl)-3-methoxy-2H-furan-5-on
28	88746	11.83	22896	Phenol, 2-methoxy-3-(2-propenyl)-
29	2991048	13.494	163570	n-Propyl heptyl ether
30	631976	14.016	28527	3,4-Altrosan
31	382264	15.507	85158	Diethyl Phthalate
32	572176	15.65	72728	Phenol, 2-(1,1,3,3-tetramethylbutyl)-
33	2014790	15.997	97573	o-Acetyl-L-serine
34	1401844	16.311	107777	3-Deoxy-d-mannoic lactone

**Table 10:** Percentage of inhibition of 1,4,7,10,13,16-hexaoxacyclononadecane of Cq

S. No	Tested Sample Concentration (μg/ml)	Percentage of Inhibition (in Triplicate)			% Inhibition (Mean ± SD)
						1.	Ascorbic acid	74.5679	74.1975	73.7654	74.18 ± 0.40
						2.	500 μg/ml	61.4198	61.1111	60.9259	61.15 ± 0.25
						3.	250 μg/ml	60.6173	60.4321	60.1852	60.41 ± 0.22
4.	100 μg/ml	58.9506	58.7037	57.5926	58.41 ± 0.71
5.	50 μg/ml	57.284	56.0494	55.1235	56.15 ± 1.10
6.	10 μg/ml	54.9383	54.3827	54.0741	54.46 ± 0.44

**Table 11:** Percentage of inhibition of 3-deoxy-d-mannoic lactone of As

S. No	Tested Sample Concentration (μg/ml)	Percentage of Inhibition (in Triplicate)			% Inhibition (Mean ± SD)
						1.	Ascorbic acid	74.5668	74.0099	74.1955	74.25 ± 0.29
						2.	500 μg/ml	65.9653	64.9134	64.7277	65.20 ± 0.65
						3.	250 μg/ml	64.4802	64.1089	63.552	64.04 ± 0.47
4.	100 μg/ml	63.4901	63.2426	62.1287	62.95 ± 0.72
5.	50 μg/ml	57.9827	57.4257	59.1584	58.18 ± 0.89
6.	10 μg/ml	57.302	56.6213	56.4975	56.80 ± 0.44
